# Comparison of rapid vs in-depth qualitative analytic methods from a process evaluation of academic detailing in the Veterans Health Administration

**DOI:** 10.1186/s13012-019-0853-y

**Published:** 2019-02-01

**Authors:** Randall C. Gale, Justina Wu, Taryn Erhardt, Mark Bounthavong, Caitlin M. Reardon, Laura J. Damschroder, Amanda M. Midboe

**Affiliations:** 1 Center for Innovation to Implementation (Ci2i), VA Palo Alto Health Care System, Stanford University, 795 Willow Rd, Menlo Park, CA 94025 USA; 2 Veterans Health Administration, Pharmacy Benefits Management, Academic Detailing Service, Seattle, WA USA; 3 VA Ann Arbor Center for Clinical Management Research, 2215 Fuller Rd (152), Ann Arbor, 48113-0170 MI USA

**Keywords:** CFIR, Implementation framework, Qualitative methods, Rapid analysis, Academic detailing, Veterans

## Abstract

**Background:**

It is challenging to conduct and quickly disseminate findings from in-depth qualitative analyses, which can impede timely implementation of interventions because of its time-consuming methods. To better understand tradeoffs between the need for actionable results and scientific rigor, we present our method for conducting a framework-guided rapid analysis (RA) and a comparison of these findings to an in-depth analysis of interview transcripts.

**Methods:**

Set within the context of an evaluation of a successful academic detailing (AD) program for opioid prescribing in the Veterans Health Administration, we developed interview guides informed by the Consolidated Framework for Implementation Research (CFIR) and interviewed 10 academic detailers (clinical pharmacists) and 20 primary care providers to elicit detail about successful features of the program. For the RA, verbatim transcripts were summarized using a structured template (based on CFIR); summaries were subsequently consolidated into matrices by participant type to identify aspects of the program that worked well and ways to facilitate implementation elsewhere. For comparison purposes, we later conducted an in-depth analysis of the transcripts. We described our RA approach and qualitatively compared the RA and deductive in-depth analysis with respect to consistency of themes and resource intensity.

**Results:**

Integrating the CFIR throughout the RA and in-depth analysis was helpful for providing structure and consistency across both analyses. Findings from the two analyses were consistent. The most frequently coded constructs from the in-depth analysis aligned well with themes from the RA, and the latter methods were sufficient and appropriate for addressing the primary evaluation goals. Our approach to RA was less resource-intensive than the in-depth analysis, allowing for timely dissemination of findings to our operations partner that could be integrated into ongoing implementation.

**Conclusions:**

In-depth analyses can be resource-intensive. If consistent with project needs (e.g., to quickly produce information to inform ongoing implementation or to comply with a policy mandate), it is reasonable to consider using RA, especially when faced with resource constraints. Our RA provided valid findings in a short timeframe, enabling identification of actionable suggestions for our operations partner.

**Electronic supplementary material:**

The online version of this article (10.1186/s13012-019-0853-y) contains supplementary material, which is available to authorized users.

## Background

The slow pace of healthcare research has been cited as a contributing factor to the dissemination of less relevant or even obsolete findings, resulting in the call for more flexible and rapid research designs [1]. Implementation science has been defined as “the scientific study of methods to promote the systematic uptake of research findings and other evidence-based practices into routine practice” [2]. Qualitative methods are important tools at the disposal of implementation scientists not only because they can be adapted to a specific implementation setting but also because they provide the ability to explore and understand in detail how well different implementation components work together. With the aim of informing and improving the quality and effectiveness of health care service delivery, implementation science is germane not only to clinicians but also to patients, payers, and policymakers. Given these important aims, qualitative analytic methods that provide for the timely evaluation, identification, and dissemination of critical intervention components while maintaining scientific rigor are needed [3].

Considerations for the use of rapid evaluation methods have been enumerated in the literature, including the examination of their value in producing actionable information to planners and decision-makers [4]. Additionally, several studies outlining different approaches to conducting rapid qualitative evaluations or assessments have been described in the literature [5–9]. Beebe defines rapid research as “research designed to address the need for cost-effective and timely results in rapidly changing situations,” [10] and others describe the use of visual displays (e.g., matrices) to assemble data in a succinct manner for display and to assist in drawing conclusions [11]. Other literature has also addressed implementers’ need for actionable feedback to guide timely integration within the context of an informatics intervention. For example, one group of researchers described the development and refinement of a rapid assessment process during which they collected and analyzed field notes, direct observation, and interview data to develop case studies for comparative analysis. Ultimately, this group was able to provide their sponsors with useful feedback in a short amount of time [12].

However, rapid analyses are not without limitations. Some groups have illustrated the challenges around maintaining trustworthiness given the rapid pace of the analyses conducted [5]. Other literature has reported a heavy workload and logistical burden on project researchers because of the compressed timeline of rapid analyses [12].

### Context and aim of the study

This study was conducted as part of a 1-year process evaluation of a successfully implemented academic detailing (AD) program to improve opioid prescribing in the Veterans Health Administration’s (VA) Sierra Pacific regional network (described in detail elsewhere) [13]. This paper aims to (1) describe our approach to conducting a rapid analysis (RA), (2) assess the consistency of our findings from the RA, and (3) discuss resource intensity of RA versus in-depth analysis of transcripts from semi-structured interviews. For the purpose of this comparison, we define “consistency” as the extent to which findings were similar across methods (i.e., in comparison to the in-depth analysis) [14], and “resource intensity” as the level of effort and amount of time needed to complete the analyses (including training in using specialized software).

In brief, AD is an evidence-based outreach strategy modeled after the pharmaceutical industry technique of meeting with and educating providers about changing their prescribing practices to be in line with evidence [3–5]. This intervention relies on face-to-face education sessions and utilization of behavior change techniques to motivate voluntary change among providers exposed to AD. Within the VA, AD has been effective in improving naloxone and opioid prescribing practices [6–11].

In collaboration with our operations partner (the Sierra Pacific Network’s Pharmacy Benefits Management), we conducted a “rapid” process evaluation guided by the Consolidated Framework for Implementation Research (CFIR); the evaluation focused on identifying (1) aspects of the Sierra Pacific network’s implementation that worked well and (2) actionable recommendations for implementation in other regional networks. “Rapid” was defined as completion of the evaluation (from design to dissemination) within 12 months. An abbreviated timeline was necessary because of time-limited funding, and a policy mandating implementation of AD programs in the remaining VA regional networks within a 6 month time period [15]. After completion of the RA, we conducted an in-depth, deductive analysis of the data using the CFIR to code transcripts [16]. The in-depth analysis is line with content analysis using a directed approach [17].

Herein, we describe and compare the two analytic methods (rapid and in-depth) used. The methods provide alternative approaches to working with qualitative data while remaining grounded in a well-established implementation framework.

## Methods

Methods for participant recruitment, data collection, and establishing trustworthiness for the process evaluation are detailed in a separate publication [13]. In short, we conducted in-person and telephone semi-structured interviews with regional academic detailers and primary care providers (including 15 physicians, 3 advanced practice nurses, and 2 psychologists integrated into primary care) who had and had not received AD between 10/1/2014 and 9/30/2015. Interviews were recorded and professionally transcribed. We conducted a CFIR-informed RA to group descriptions of implementation lessons learned into distinct categories and to provide immediate feedback to our operations partner. We later performed a deductive in-depth analysis also using the CFIR.

The core evaluation team was led by a research psychologist (AMM). The qualitative lead (RCG) has a doctorate in public health. The remainder of the analytic team included a Doctor of Pharmacy (MB) and two personnel with training in public health (JW, TE).

The evaluation met the definition of quality improvement and was determined by the Institutional Review Board of record, Stanford University, to be non-human subjects research.

### Interview guide development and data collection

The CFIR was selected a priori to guide identification of facilitators (what worked well) and barriers (opportunities for improving implementation) for the overall process evaluation. The CFIR is a meta-theoretical framework particularly well-suited to our evaluation, given its flexibility and adaptability with respect to identifying key influences on implementation from the perspective of multiple stakeholders. Interview guides were therefore designed to encompass key constructs of the five CFIR domains (i.e., intervention characteristics, outer setting, inner setting, characteristics of individuals, and process). With input from our operations partner, we iteratively developed separate but related interview guides based on participant role (Table 1): academic detailer or provider (inclusive of providers who had detailing sessions and providers who had been offered sessions but had not engaged). (See Additional file 1 for a copy of the academic detailer interview guide.) Questions were open-ended, designed to elicit detailed descriptions from participants related to implementation and their perceived effectiveness of AD. From the perspective of delivering AD, we aimed to identify training, outreach, and engagement strategies. From the perspective of providers receiving academic detailing, we aimed to identify gaps in knowledge about AD as well as best practices for outreach and engagement. Individual questions were mapped to CFIR domains and constructs to ensure we were probing for the information which the evaluation team and our operations partner considered to be the most relevant information from across the framework.

**Table 1:** Example interview guide questions and related CFIR domains/constructs

Participant type	Question	CFIR domain	CFIR construct
Academic detailer	How do you engage providers in academic detailing?	Process	Engaging
	What kinds of training materials have you used? (e.g., in-person seminars, web sites, journal articles, SharePoint, teleconferences, other items)	Intervention characteristics	Design quality and packaging
Detailed provider	How do you think academic detailing compares to other programs intended to improve prescribing practices? What are the advantages/disadvantages of this program compared to others? Are there other programs that would be more useful?	Intervention characteristics	Relative advantage
	How confident are you that you can make the changes recommended by academic detailers or similar folks?	Characteristics of individuals	Individual stage of change
Not detailed provider	How supportive or not is your medical center in encouraging your participation in programs like academic detailing?	Inner setting	Implementation climate
	What kind of information or evidence are you aware of that shows whether academic detailing works?	Intervention characteristics	Evidence strength and quality

*CFIR* Consolidated Framework for Implementation Research

Interview guides were pilot tested with two academic detailers and two providers to identify areas for refinement based on feedback prior to initiating data collection. This step also served as a training opportunity for analysts lacking prior interview experience.

### Rapid analysis: step 1, summarize individual transcripts

**Fig. 1:**
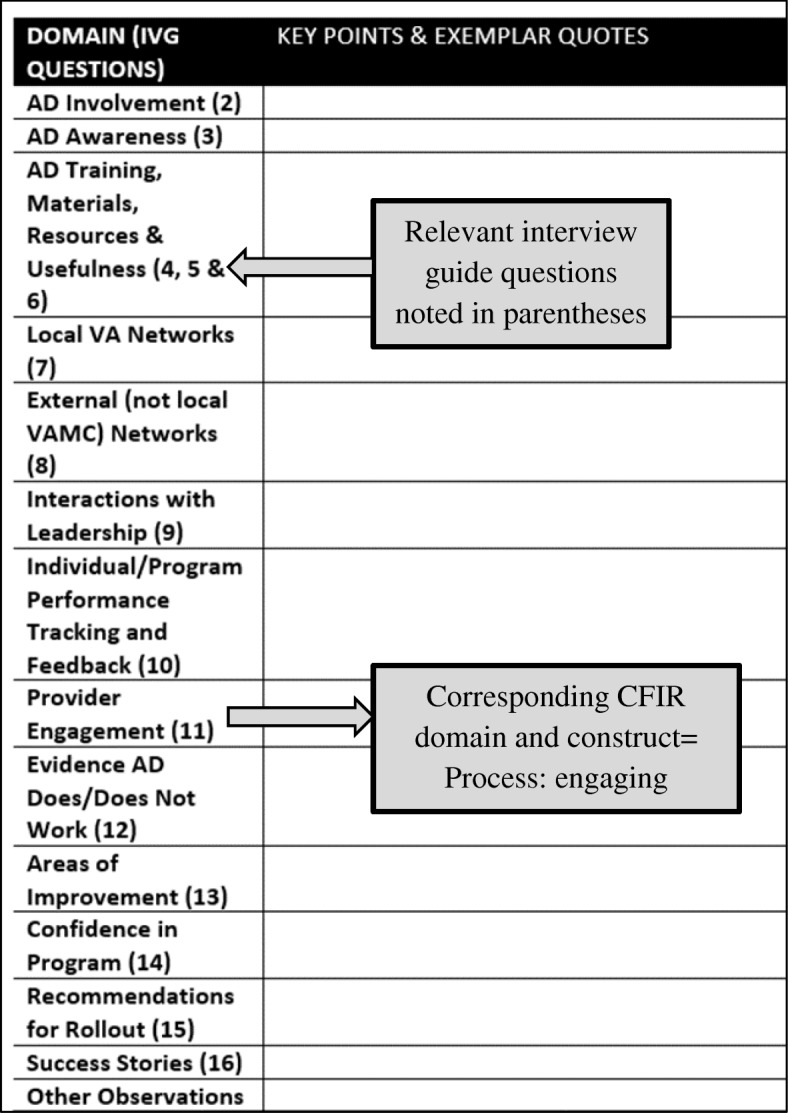
(Rapid analytic step 1) Templated summary table used to summarize each interview transcript. Example from academic detailer interview summary table; similar tables were generated for detailed and not detailed providers. *IVG* interview guide, *AD* academic detailer/detailing, *VA* Veterans Affairs, *VAMC* Veterans Affairs Medical Center, *CFIR* Consolidated Framework for Implementation Research

The first analytic step of the RA involved developing a templated summary table which the evaluation team could populate with data extracted from interview transcripts, including illustrative quotes (Fig. 1). To construct the templated summary table, the qualitative lead (RCG) took each CFIR-based interview guide and constructed a table in MS Word. The first column of the table identified pre-specified “domains” based on the CFIR-informed interview guides and key questions identified by our operations partner. The second column was used to summarize key points from the interviews and to capture illustrative quotes. The draft summary table was reviewed and modified based on feedback from the evaluation team lead (AMM), and after being tested by the analytic team (RCG, JW, TE) with a single transcript. Testing was repeated with a second transcript after incorporating modifications.

Key domains in the summary table encompassed aspects of implementation such as best practices for interacting with and leveraging networks to engage providers in AD appointments (academic detailer perspective), and the extent to which there is a sufficient body of evidence supporting the implementation of the AD program (provider perspective). To provide guidance to analysts completing the summaries, the table identified corresponding interview guide questions that would likely have prompted participants to address the specified aspect (domain) of implementation.

Using the templated summary table, the analytic team (JW, TE) generated a summary for each interview transcript. The qualitative lead (RCG) conducted a secondary review of the summaries and discussed with the analysts to ensure consistency in the data being recorded across interviews. This secondary review and discussions resulted in some revision to the content of a small number of summaries though the overall consistency and quality of the summaries was satisfactory.

### Rapid analysis: step 2, consolidate transcript summaries by participant type

**Fig. 2:**
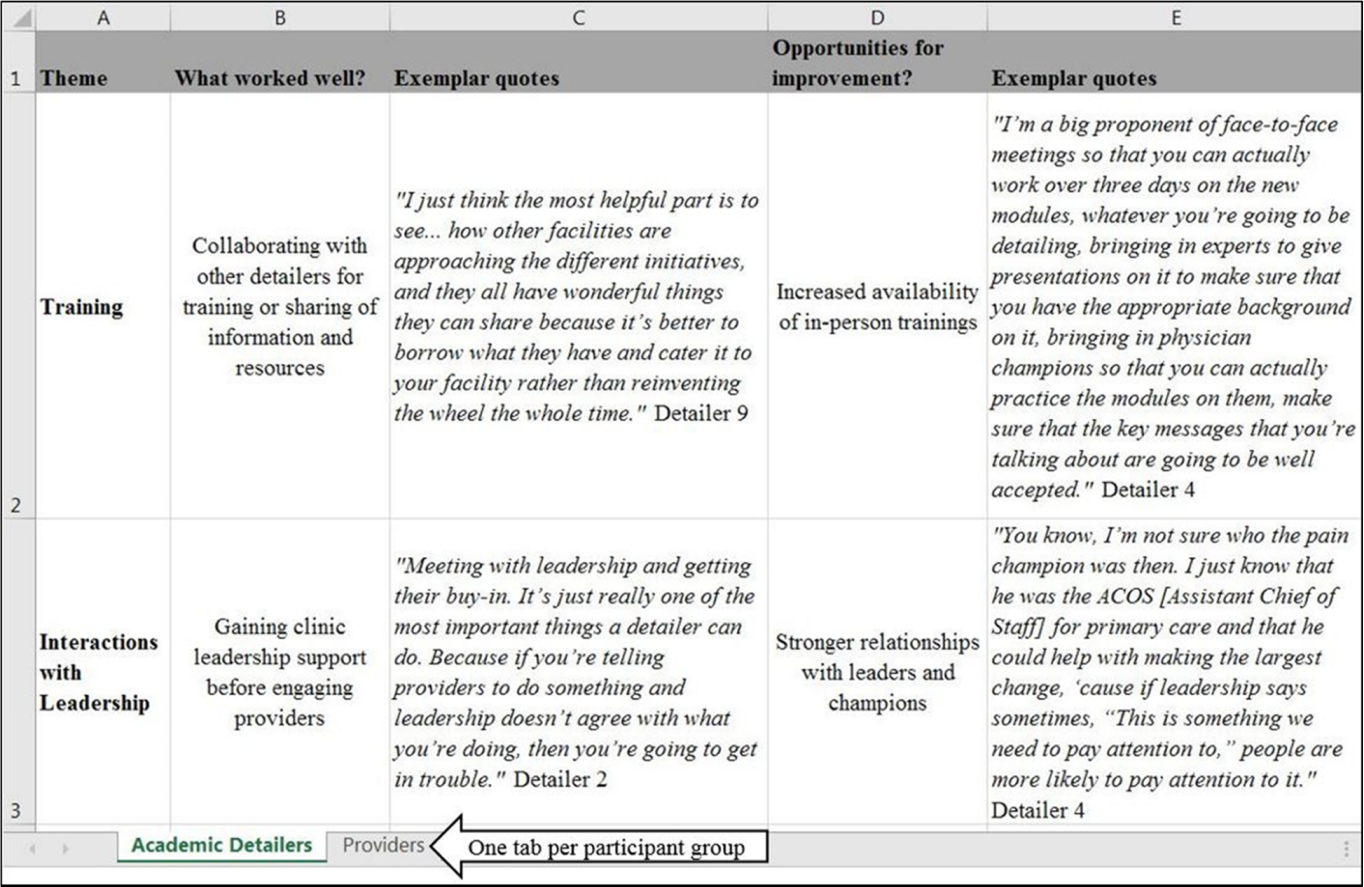
(Rapid analytic step 2) MS Excel matrix by participant type for identifying themes, sorting, and visual display; populated using information from templated summary table

The second analytic step of the RA involved consolidating the 30 interview summaries (step 1) by participant type (i.e., academic detailers, detailed and not detailed providers) for visual display, to identify commonly occurring themes, and to allow comparison across groups. To do this, we used information from the transcript summary tables to create a new matrix in MS Excel (one tab per participant group) (Fig. 2). The matrix was designed to capture several pieces of data:

1. Broad themes or categories. The initial set of themes or categories were derived from the domains represented in step 1 of the RA (e.g., the role of training or interacting with leadership).
2. Within each theme, a brief descriptor (sub-theme) of what participants reported as working well (e.g., sharing resources amongst academic detailers as part of training) and what they reported as gaps or aspects of implementation that were not working well (referred to as “opportunities for improvement”).
3. Supporting quotes (the evidence) to support the identified best practices and opportunities for improvement.

Individual transcript summaries (from step 1) were reviewed and used to populate the MS Excel matrix by participant type. During a series of in-person and virtual meetings, the analytic team collaboratively and iteratively reviewed, discussed, and sorted the data to refine the initial list of themes and sub-themes and to highlight the most salient quotes. For example, one theme to emerge from the academic detailer interviews related to the extent to which fidelity to the “pure” AD model was a critical component of implementing the intervention. Broad themes were not limited to a single sub-theme. For example, in addition to *sharing of resources amongst academic detailers*, the training theme had separate sub-themes for *training in motivational interviewing techniques* and *the incorporation of role playing into training sessions*.

Data from this step of the RA were used to create presentations and deliver a report to our operations partner with recommendations for optimizing implementation of the AD program across the VA.

### In-depth analysis

After successfully delivering a product to our operations partner, we returned to the verbatim transcripts and conducted line-by-line coding using the CFIR approximately 6 months after the original RA. The coding team included three of the original RA coders (RCG, JW, TE) and one new coder (MB), who was added to assist with the more time-intensive analysis. To begin, the qualitative lead (RCG) modified the publicly available CFIR Codebook Template [18] to address the evaluation aims (Table 2). This included adding language and examples specific to the AD program. Because the interview guides were developed using the CFIR, potentially relevant questions from the interview guides were provided as examples of when during the interviews participants were likely to have discussed something related to a code. Analysts were given instructions to use the sample questions only as a guide during coding and to not limit themselves to a specific code based on the interview question. Rather, they were told to base codes on participants’ responses to the questions. The draft CFIR codebook was reviewed by the project lead (AMM) as well as an expert (CMR) in the use and application of CFIR in evaluation work. Changes to the codebook were incorporated based on their feedback.

**Table 2:** Excerpts from CFIR codebook for the “Characteristics of Individuals” domain

Characteristics of individuals	Definition	Related interview guide questions [AD = academic detailer guide DP = detailed provider guide NDP = not detailed provider guide]	Example
1. Construct: Knowledge and beliefs about the innovation [Short code: knowledge-belief about academic detailing]	Individuals’ attitudes toward and value placed on the academic detailing program, as well as familiarity with facts, truths, and principles related to it. May double code passages related to familiarity with evidence about the innovation to “Evidence Strength and Quality.”	AD: How did you become involved with the academic detailing program? NDP: Can you tell me a bit about your understanding of what the academic detailing program for opioid prescribing is about? NDP: How did you first learn about the academic detailing program for opioid prescribing? NDP: Why do you think you were offered an appointment with an academic detailer?	Feelings about the academic detailing program and the extent to which they find it to be a valuable and worthwhile offering.
2. Construct: Self-efficacy [Short code: self-efficacy]	Individuals’ belief in their own capabilities to complete courses of action to achieve implementation goals. May be appropriate to double code to “Compatibility.”	DP: Why do you think you have or have not been offered an appointment? DP: How confident are you that you can make the changes recommended by academic detailers or similar folks. DP & NDP: What forms of continuing medical education do you find most useful or more useful than academic detailing?	The extent to which academic detailers feel they are capable of effectively functioning in this role and the extent to which providers feel they can adopt the behavior change promoted by academic detailing.

*CFIR* Consolidated Framework for Implementation Research

To validate the codebook and train the analytic team prior to coding transcripts, the team (RCG, JW, MB, TE) independently coded a single academic detailer transcript as well as a single provider transcript. The team then met through a series of teleconferences to review the coded transcripts. Each coded passage in the transcripts was reviewed and discussed until consensus was achieved. Where the codebook was unclear or needed additional examples, modifications were identified and incorporated. This step of consensus coding was repeated with a second set of academic detailer and provider transcripts. The analytic team split in pairs to code the remaining transcripts. Pairs met to discuss and resolve disagreements. All CFIR coding was completed using the Atlas.ti (version 7.5) [19] qualitative data analysis software. The team members (JW, MB, TE) received training from the qualitative lead (RCG) on use of this software.

Once initial coding was complete, we generated frequencies for each of the codes across the project as well as by interview type (i.e., academic detailers and providers). The team collectively reviewed passages coded with infrequently used codes to discuss and verify the appropriateness of the applied code. Code frequencies and the strength (quality) of the coded passages were used to identify key themes from the in-depth analysis. Findings from these analyses are presented in a separate publication [13].

### Consistency

To explore whether findings from the in-depth analysis were consistent with findings from the RA, themes from the RA were mapped to the CFIR constructs and domains identified during the in-depth analysis (Table 3).

**Table 3:** Relationship between rapid analysis themes and CFIR constructs, by participant type

Rapid analysis theme	CFIR construct	CFIR domain	Exemplar quote from CFIR coding
Academic detailers^a^			
A. Detailer training	➔ Access to knowledge-information	Inner setting	“I think they are [leadership] definitely very receptive, and I think a part of that is because it started as a pilot program, so it’s been around here at least three years. They have gotten to see a lot of the good that’s been able to come out of it. Leadership is very receptive, providers are not so much. But definitely like our ACOS [Assistant Chief of Staff] of primary care, totally on board, and our mental health leadership are definitely on board.” Detailer 5
B. Strong networks	➔ Networks-communication		
C. Performance tracking	➔ Goals-feedback		
D. Leadership support^a^	➔ Leadership engagement		
E. Detailer-provider engagement^a^	➔ Adaptability	Intervention characteristics	“I think one of the nice things that our program manager allowed us to do was to tailor our detailing to the needs of our facilities and our style. We were encouraged to develop our own style… I felt like my providers needed to be, like things needed to be maybe said in a more roundabout way, which probably wasn’t the most efficient.” Detailer 3
D. Leadership support^a^	➔ Engaging internal implementation leaders ➔ Engaging opinion leaders	Process	“Gaining leadership support, so meeting with, taking the time to meet with whoever is Service Chief or even Chief of Staff or Director to make sure they are on board. Because if you do not have leadership behind you, any time you spend with physicians can easily be disregarded and nobody else is kind of driving that same message.” Detailer 7
E. Detailer-provider engagement^a^	➔ Engaging key stakeholders		
Providers^b^			
A. Performance tracking	➔ Goals-feedback	Inner setting	“The leadership, all are supportive. I mean, if we have somebody, we take them off medications, it ends up going to the quad, and they are supportive if we are not going to be giving somebody their narcotics for a specific reason.” Provider 1–4
B. Leadership support	➔ Leadership engagement ➔ Available resources		
C. Materials and resources	➔ Design quality-packaging	Intervention characteristics	“Yeah, I – I think specifically the little binder one was the one [materials] I sort of use the most. I work with some of our residents in internal medicine and I have been able to sort of like use them to kind of hand out things that they can take home and as far as like, ‘Here’s what I look at. You should look at this too.’” Provider 1–5
D. Perceived value^b^	➔ Design quality-packaging		
D. Perceived value^b^	➔ Knowledge-beliefs about the intervention	Characteristics of individuals	“I think it’s an effective way. I think it is. I think sort of it’s very human to be face-to-face with someone talking.” Provider 2–1
E. Motivating behavior change^b^	➔ Self-efficacy		
D. Perceived value^b^	➔ Engaging key stakeholders	Process	“She contacted me over email and the initial appointment that we had, which I think was before the Opioid Safety Initiative, she actually made the trip up to [the outpatient clinic] to meet me and primary care folks in person, and then the Opioid Safety Initiative meetings have mainly been by phone and with supplemental information that she has sent me by email.” Provider 2–2
E. Motivating behavior change^b^	➔ Engaging key stakeholders		
F. Detailer-provider engagement	➔ Engaging key stakeholders ➔ Engaging internal implementation leaders ➔ Executing		

*CFIR* Consolidated Framework for Implementation Research

^a^Academic detailer rapid analysis theme related to multiple CFIR constructs/domains

^b^Provider rapid analysis theme related to multiple CFIR constructs/domains

### Resource intensity

**Fig. 3:**
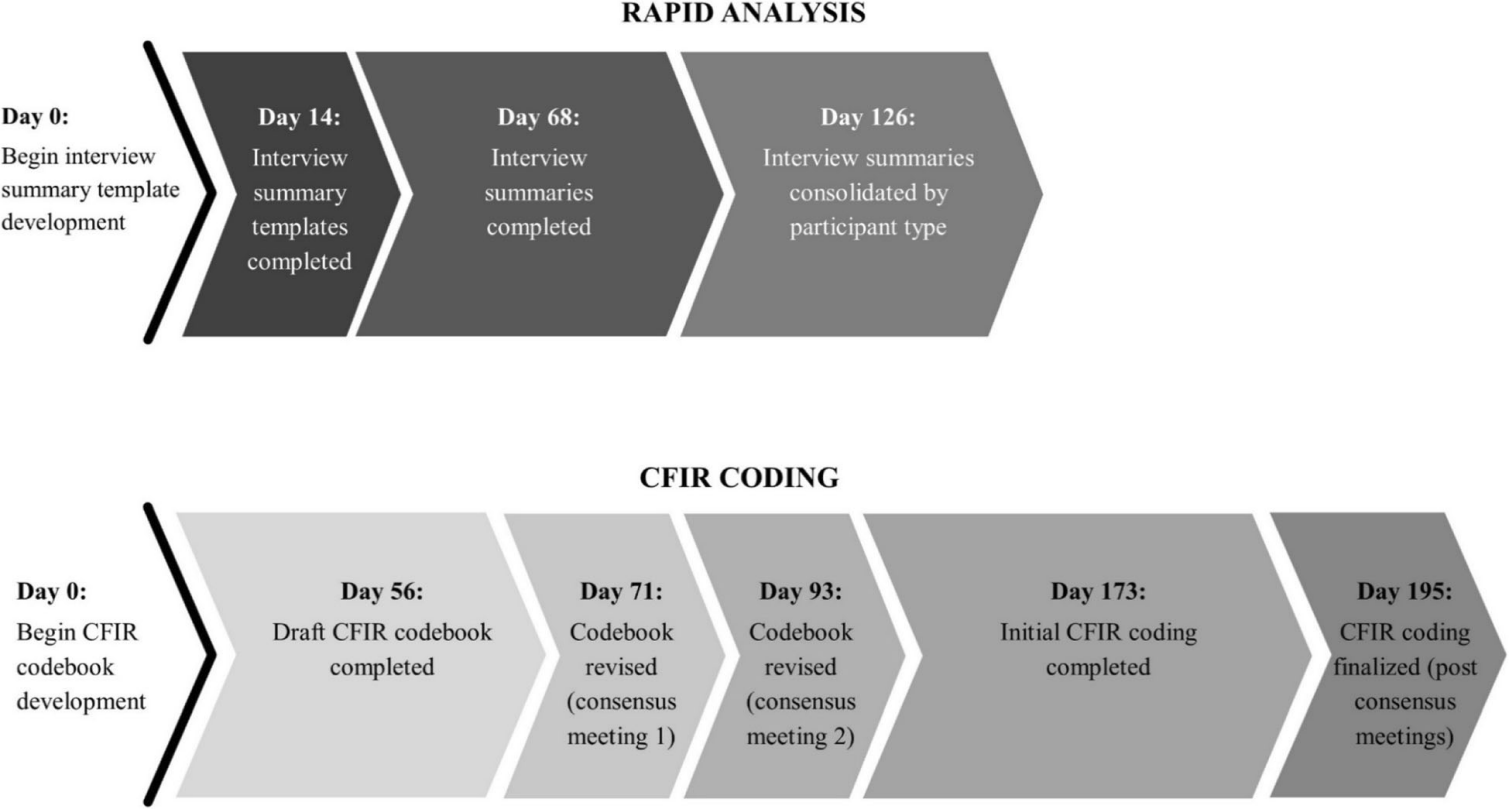
Timeline for conducting rapid and in-depth analysis. Some transcript coding took place as part of CFIR codebook development (i.e., the first 93 days). *CFIR* Consolidated Framework for Implementation Research

We maintained data related to how long it took to complete key steps for both the rapid and in-depth analyses. Data were compared to determine which analytic method required more training and time to complete (Fig. 3).

## Results

### Parent process evaluation

Data collection for the process evaluation took place between February and May 2016. Thirty in-person and telephone semi-structured interviews with regional academic detailers (all clinical pharmacists) (*n* = 10) and providers (*n* = 20) were conducted. Thirteen of the providers had received AD; 7 had not. Providers included physicians (*n* = 15), advanced practice practitioners (*n* = 3), and clinical psychologists (*n* = 2). Transcripts from all 30 interviews served as the primary data source for the methods comparison described herein. Additional details about the findings from the evaluation of academic detailing can be found in a separate publication [13].

### Consistency of findings

We defined “consistency” as the extent to which findings were similar across the two analytic methods. Our qualitative comparison (mapping) of findings from the RA to the primary domains and constructs identified during in-depth analysis did not reveal any significant information gaps. As an example, the CFIR domain “Outer Setting” refers to aspects of implementation like peer pressure, external mandates, and public benchmarking. We did not identify any aspects of this domain in either our rapid or in-depth analysis of transcripts. In fact, themes identified during the RA mapped well to constructs identified during in-depth analysis (Table 3).

Most of the CFIR-coded passages fell within the *Inner Setting*, *Intervention Characteristics*, and *Process* domains for both detailer and provider interviews. Constructs within the *Characteristics of Individuals* domain were also coded for providers. The five themes identified through RA of academic detailer interviews were aligned with eight CFIR constructs within three unique domains (inner setting, intervention characteristics, and process). Similarly, the six themes identified during the RA aligned with nine CFIR constructs within four unique domains. For example, within the CFIR domain, *Inner Setting*, *Leadership Engagement*, and *Available Resources* aligned with the RA theme “Leadership Support.”

Both rapid and in-depth analysis revealed several similar best practices related to implementation of the AD program including:

1. Allowing flexibility for detailers to adapt recruitment and engagement strategies to individual medical facility and provider context.
2. Increasing the frequency of and access to academic detailer training like motivational interviewing, and providing opportunities to practice new skills (e.g., through role play).
3. Building and leveraging relationships with leadership, primary care pain champions, and committees, to support and promote the program, educate providers and other leaders, and help secure time for staff to interact with academic detailers.

### Resource intensity

Comparing the timeline for completion of the RA to completion of the in-depth analysis revealed that it took 69 days or approximately 10 weeks (Fig. 3) longer to complete the in-depth analysis. The time investment required to complete line-by-line coding depends on many factors including previous coding experience, previous experience using qualitative data analysis software, and previous experience using CFIR. The calculation of 69 additional days to complete the in-depth analysis for this comparison is likely conservative as the analytic team had previous experience working with the transcripts to complete the RA. The team also spent time refining the list of themes identified during the RA, which provided some initial insights with respect to what CFIR codes might be applicable to a given passage. The CFIR itself was incorporated throughout the project (evaluation design, interview guide development, summary template development), likely contributing to the ability to complete the in-depth analysis in a relatively short amount of time.

## Discussion

The goals of this paper were to describe our approach to conducting a CFIR-informed RA, assess the consistency of findings from our RA in comparison to an in-depth analysis of the same data, and compare resource intensity of the two analytic approaches. Overall, we found RA to be sufficient for providing our operations partner with actionable findings and recommendations, which was necessary given the relatively short timeline included in the policy mandate for implementation of AD programs throughout the VA.

With respect to consistency of our RA and in-depth analysis findings [14], themes from the RA were well-aligned with the CFIR domains and constructs from the in-depth analysis. Considering the CFIR was embedded throughout the evaluation, including the design of interview guides and indirectly in development of the summary tables, these findings are not entirely unexpected. Upon further reflection, we could have elected to more explicitly incorporate the CFIR constructs into the RA summary tables rather than indirectly through the interview guides, and this may have made RA even faster. This would still be considered a rapid analytic approach, but would have carried the CFIR more transparently throughout the RA portion of the project. Depending on the anticipated uses of similar evaluation data, this may further streamline the method.

Given the complexity of the CFIR (i.e., multiple constructs per domain), rapid analytic methods like ours may be helpful when working with large numbers of interviews where line-by-line coding and analysis may not be possible, and/or when evaluating highly complex interventions where one needs to quickly identify key aspects of implementation. However, careful consideration should be taken prior to adopting this approach to limit the potential for bias and to limit the potential for providing an overly narrow interpretation of the data. It is important to keep in mind that the combination of the strength and frequency of qualitative comments is what helps us understand their relative importance and contributions to our research [20], regardless of whether you are using a rapid or in-depth analytic approach.

There are some important tradeoffs to consider when electing to conduct a RA like ours. One such tradeoff may relate to the ability to rate constructs (e.g., constructs from a framework like the CFIR) and relate those ratings to implementation outcomes. This rating technique was described by Damschroder and Lowery [21, 22] in their application of quantitative ratings to CFIR constructs as part of a CFIR-guided evaluation of a weight management program; it applies a valence (barrier or facilitator to implementation) and strength (weak or strong barrier or facilitator to implementation) rating to each construct. In that project, they found that some constructs distinguished between facilities with low and high implementation success. While RA may be well-suited for providing rapid, relevant feedback to stakeholders, including determining valence of a construct, it may be more challenging to determine strength of a construct, given the high-level data used in RA. A related tradeoff of our approach to RA is that it limits one’s ability to compare findings across projects unless findings are mapped to a framework; because the CFIR provides a consistent taxonomy, it is easier to compare findings between different projects that explicitly used the CFIR to analyze qualitative data.

Because we were interested in assessing the resource intensity of the RA as compared to the in-depth analysis, we maintained a timeline for key steps in each analytic approach. Although we did not track the number of hours spent by each analyst working on the two analyses, we were able to use our timeline to make some broad comparisons between the different analytic approaches. Our in-depth analysis took considerably (69 days) longer than the RA to complete. In their publication, Neal et al. compared line-by-line coding of transcripts to the analysis of field notes [23]. Based on their experience and review of the literature, they assert that line-by-line coding of verbatim transcripts allows for the retention of a high-level of interview detail; however, nonverbal details or cues are not retained at the same level of detail. According to their summary, line-by-line coding is a relatively slow process, which is consistent with our experience.

Neal and colleagues [23] did not specifically mention training and expense in their comparison of different analytic methods. We found that line-by-line coding required a substantial up-front investment in training—analysts must be trained on use of a codebook as well as use of qualitative data analytic software. Intensive training for the in-depth analysis was needed even though (except for one analyst) the team that conducted the in-depth analysis was the same as the team that conducted the RA. Had our team composition varied drastically, training for the in-depth analysis could have taken even longer.

Establishing inter-rater reliability among analysts (i.e., through consensus coding) was more time intensive than establishing consistency in how transcripts were summarized for the RA. For the most part, training for the RA was limited to learning how to use and populate the templates (i.e., what data goes where). Finally, costs associated with the different analytic methods should not be overlooked. Assuming a project is working with verbatim transcripts, there are fixed costs associated with transcription. Modifiable costs based on the chosen analytic approach include the cost of qualitative data analytic software (high) or word processing software (low) and labor. With respect to labor, line-by-line coding of verbatim transcripts required considerably more analytic time than summarizing transcripts.

*Validity* in qualitative research has been defined as the “appropriateness of the [selected] tools, processes, and data” [14]. We did not set out to assess the validity of our RA method prior to data collection, as the decision to apply this technique was made pragmatically in concert with our operations partner to meet their need for timely, valid, and actionable findings. Others considering a similar analytic method should weigh the tradeoffs when considering whether the method is appropriate for and sufficient to answer their research question including the potential for introducing bias when using a rapid analytic method such as ours where summarizing themes or concepts may require more subjective “interpretation” of the data than is needed when applying a structured framework like the CFIR as part of an in-depth analysis.

Findings from this comparison of rapid and in-depth analytic methods must be interpreted with caution. Estimates of time to completion are specific to the context and complexity of this project, the implementation setting, the evaluation aims, team experience and composition, level of funding, and other competing priorities. None of the evaluation team members were dedicated 100% to this project. Being able to dedicate larger amounts of time on any given day, especially when it comes to analyzing (summarizing or coding) the data, likely would have expedited the process, although some early activities like participant recruitment and interview scheduling are fixed and could not have taken place much faster. It is important to note that time spent developing interview guides was not included in estimates of time to completion. Additionally, because the interview guide questions and summary tables were mapped to CFIR, team members were already exposed to CFIR constructs and may have completed the CFIR coding faster than a CFIR-naive group of analysts would have.

Staffing, funding, and other resource constraints make it challenging to rapidly complete and generate valid findings from research and evaluation projects. Delays can impede implementation of innovative programs or interventions when data are needed to monitor, modify, or scale-up, or when policy changes necessitate the need for timely feedback. Our team was charged with providing rapid feedback to implementers of a successful AD program in one VA regional network for dissemination across the VA. To accomplish this, we successfully applied the use of a rapid analytic method.

## Conclusions

Achieving balance between the need for actionable results and scientific rigor is challenging. The use of rapid analytic methods for the analysis of data from a process evaluation of a successful AD program proved to be adequate for providing our operations partner with actionable suggestions in a relatively short timeframe.

Ultimately, themes identified during the RA mapped well to the CFIR constructs. This approach to analyzing qualitative data provides an example of alternative methods to working with qualitative data that are still guided by well-established implementation frameworks. It is reasonable to consider RA methods informed by frameworks like the CFIR in rapid-cycle or resource poor projects, or when working to provide “real-time” data to policy and operation partners. However, tradeoffs like the ability to rate the data and compare findings with other studies using the same taxonomy (e.g., CFIR) must also be weighed when making these analytic decisions.

Although this paper is focused primarily on analysis of data, it is important to remember that it is only one component of a qualitative study, albeit a critical one. To increase the likelihood of credibility, dependability, and trustworthiness of data, there are several decisions that should be considered when conducting qualitative evaluations, from design through execution [24]. Analysis considerations should be assessed alongside other study priorities, such as available resources or broader theoretical methodology, to best meet the goals of the evaluation being conducted.

## Additional file

Additional file 1: Academic detailer interview guide questions. (DOCX 39 kb)

## Abbreviations

AD: Academic detailing
CFIR: Consolidated Framework for Implementation Research
IVG: Interview guide
RA: Rapid analysis
VA: Veterans Health Administration
VAMC: Veterans Affairs Medical Center
