# Supplementary material for: Comparison of rapid vs in-depth qualitative analytic methods from a process evaluation of academic detailing in the Veterans Health Administration
Source: Implement Sci. 2019 Feb 1;14:11. doi: 10.1186/s13012-019-0853-y (PMC6359833; doi:10.1186/s13012-019-0853-y)
Supplement: Supplementary file 1 — Academic detailer interview guide questions. (DOCX 39 kb) [file 13012_2019_853_MOESM1_ESM.docx]

**Additional file 1**. Academic detailer interview guide questions

[Green text denotes potential CFIR domains or constructs]

**Background**

1. Can you tell me how long you’ve worked at the VA? How long have you been working as an academic detailer? [BACKGROUND & CHARACTERISTICS OF INDIVIDUALS]
   1. What is your current role in the VA?
2. How did you become involved with the academic detailing program? [CHARACTERISTICS OF INDIVIDUALS – KNOWLEDGE AND BELIEFS ABOUT THE INTERVENTION]
   1. What are your primary responsibilities as a detailer? [BACKGROUND & CHARACTERISTICS OF INDIVIDUALS]
   2. What proportion of your time do you spend detailing related to opioid safety versus other topics?
   3. How do you split your time among detailing and non-detailing responsibilities?
3. Are you aware of the policy mandating implementation of academic detailing across the VA?
   1. What is your sense of why the academic detailing program is being implemented across the VA? [INTERVENTION CHARACTERISTICS: INTERVENTION SOURCE]
   2. How do implementation/academic detailing Opioid Safety Initiative (OSI) efforts align with other organizational goals? (Facility, VISN, VA goals) [INNER SETTING – GOALS AND FEEDBACK]

**Participant Involvement with Academic Detailing**

1. Tell me a bit about the training you received as an academic detailer. [INNER SETTING – IMPLEMENTATION CLIMATE]
   1. Was the training you received adequate to prepare you for the work you do as a detailer? Why or why not?
   2. What kinds of training materials have you used? [For example: in-person seminars, web sites, journal articles, SharePoint, teleconferences, other items?] [INTERVENTION CHARACTERISTICS – DESIGN QUALITY & PACKAGING]
      1. How accessible are training materials? [Note: want to explore when and how participant accesses these materials. Was it a one-time thing or do they regularly review training materials?]
      2. How useful have the training materials been? [Note: trying to understand the quality of the materials]
         1. Which materials have been most helpful? Why?
         2. Which have been less helpful? Why?
   3. What’s worked well? Why?
   4. What’s worked less well? Why?
   5. What suggestions do you have for improving the training of **new academic detailers**? [May want to prompt that we’re interested in concrete materials and/or trainings such as motivational interviewing.] [INTERVENTION CHARACTERISTICS – ADAPTABILITY]
   6. What suggestions do you have for the ongoing training of **current academic detailers**? [INTERVENTION CHARACTERISTICS – ADAPTABILITY]
2. Beyond the training materials and resources we’ve just discussed, what additional resources do you feel you need to be an effective academic detailer?
3. There are many things you do as an academic detailer. What do you think are the elements or components that make it most effective? [INTERVENTION CHARACTERISTICS – ADAPTABILITY]

**Networks in the VA**

1. In what ways do you interact with the facility’s pain committee? Please explain.
   1. Do you engage with pain leadership at the facility or VISN level? (Probe for explanation.)
   2. What about the primary care pain champion? [Note: they may not be aware of the primary care champion or it may be the same person.]
2. Where and when do you interact with colleagues and other academic detailers about the program? [INNER SETTING – NETWORKS & COMMUNICATIONS]
   1. What is helpful about those interactions?
   2. What suggestions do you have for engaging new academic detailers and making them feel like they are part of the team?
      1. For improving knowledge about the program?
3. Where and when do you interact with academic detailing/VISN/Medical Center leadership? [INNER SETTING – NETWORKS & COMMUNICATIONS & READINESS FOR IMPLEMENTATION – LEADERSHIP ENGAGEMENT]
   1. How receptive or supportive is the facility leadership to the academic detailing? [INNER SETTING – IMPLEMENTATION CLIMATE]
   2. How receptive or supportive is the clinic leadership to the academic detailing intervention? [INNER SETTING – IMPLEMENTATION CLIMATE]
   3. What do you think would be helpful in getting more support and participation in the program? (This might be leadership, political, materials, research, etc.) [READINESS FOR IMPLEMENTATION – ACCESS TO KNOWLEDGE & INFORMATION]

**Detailer Performance and Program Feedback**

1. How are you assessed on how well you are doing as a detailer? [PROCESS – REFLECTING & EVALUATING]
   1. How do you personally track **your own** performance?
      1. Is the information helpful?
      2. What would make it more helpful?
   2. What kind of feedback do you receive on **your** performance?
      1. How often?
      2. Is that information helpful?
      3. What would make the information more helpful?
   3. What kind of information is tracked on **overall** program performance?
      1. What specific goals are tracked and how? [Probe for both individual and program performance].
   4. What recommendations do you have for streamlining the data collection process?
2. How do you engage providers in academic detailing? [PROCESS – KEY STAKEHOLDERS]
   1. What are the most effective ways of engaging them?
   2. How do you handle individuals who seem reluctant to speak with you?
   3. What suggestions do you have for new academic detailers about ways to engage providers?
   4. Other than groups we’ve discussed, what other key individuals or groups have or could be encouraged to participate in academic detailing (e.g., pharmacy leadership, mental health leadership, medical center director, etc.)?
3. What evidence are you aware of that shows if academic detailing works? [INTERVENTION CHARACTERISTICS – EVIDENCE STRENGTH & QUALITY] [Evidence = literature, research, studies, experience, etc.]
4. What aspects of the detailing program just don’t work well? How would you improve them? [INTERVENTION CHARACTERISTICS – ADAPTABILITY, TRIALABILITY]
   1. Are there other programs that would be more useful in improving opiate prescribing practices? What are they? Why do you think they would be more useful? [What makes you say there are no other more useful programs?]

**Recommendations**

1. How confident are you that the program is having the intended outcome? Why? [INTERVENTION CHARACTERISTICS – RELATIVE ADVANTAGE]
2. What recommendations do you have for other VISNs interested in implementing a similar program? [GENERAL CONTEXT]
3. Finally, can you share a story or two that you think will help others understand how successful the program has been?

**Concluding Remarks and Thank You**
